# Comparison of a Novel Ultrasonic Scaler Tip vs. Conventional Design on a Titanium Surface

**DOI:** 10.3390/ma11122345

**Published:** 2018-11-22

**Authors:** Bruna Sinjari, Gianmaria D’Addazio, Martina Bozzi, Renato Celletti, Tonino Traini, Luan Mavriqi, Sergio Caputi

**Affiliations:** 1 Department of Medical Oral and Biotechnological Sciences, University “G. d’Annunzio” of Chieti-Pescara, Via dei Vestini 31, 66100 Chieti (CH), Italy; martinadental.studio@libero.it (M.B.); renato.celletti@unich.it (R.C.); t.traini@unich.it (T.T.); scaputi@unich.it (S.C.); 2 Department of Periodontology, Albanian University, Str. Durres, 1001 Tirana, Albanian; luanmavriqi@yahoo.com

**Keywords:** titanium surface, surface topography, dental implant, in vitro

## Abstract

The aim of this in vitro study was to evaluate the alterations of a titanium surface after treatment with two different types of ultrasonic tips: conventional steel versus an innovative copper alloy silver-plated one. Twenty smooth-surface, grade IV unalloyed titanium discs were divided into two groups. The discs were ultrasonically instrumented and the scaler was connected with a loading machine. The surface morphology was examined using scanning electron microscopy (SEM) and fractal analysis of lacunarity was calculated to highlight the alteration of the surface using the two different tips. The SEM analysis showed different degrees of surface roughness between the two types of scaler tips. Moreover, these observations demonstrated that the new tip showed fewer irregularities on the disc’s surface than the conventional steel tip. The statistical and fractal analysis showed a statistically significant difference between the two groups. Surface alterations of titanium induced by the conventional ultrasonic tips were much greater than those made by copper alloy silver plated tips. The presented results suggest that the use of this new ultrasonic tip may reduce the alterations on the implant surface during its use in dental practice.

## 1. Introduction

The presence of bacteria on the implant surface may cause peri-implant mucositis (a reversible inflammation of the peri-implant tissues) or periimplantitis (a non-reversible process which causes bone loss) [1,2]. The bacteria can adhere to the titanium irregularities by establishing an inflammatory state, leading to loss of bone and thus of osseointegration. Over the years, different surface treatments have been studied to reduce or eliminate this phenomenon [3]. Several authors have shown that a rough surface is more attractive for cell growth and, in particular, for osteoblast growth [3,4]. On the other hand, the surface roughness seems to also be attractive for bacteria and increases bacterial plaque accumulation on the implant surface. For this purpose, some surface treatments with an extremely tightly controlled roughness have been developed and were demonstrated to have a good attractiveness for the cells but less for bacterial colonization, reducing the risk of peri-implantitis and contamination of the implant surface [5,6].The treatment for peri-implantitis includes decontamination of the surface exposed to biofilm in order to eliminate inflammation and to make the exposed surface biocompatible with re-osseointegration as the ultimate objective [7,8,9,10]. In patients with implant-supported prostheses, good oral hygiene with a correct mechanical instrumentation is mandatory to maintain a smooth surface favouring the re-establishment adhesion of soft tissues [11,12,13,14,15]. Several authors have investigated the tools to clean and smooth the implant surface without modifying its topography. Metallic and non-metallic instruments are used for the hygiene procedures of dental implants. In fact, conventional instruments such as sonic scalers, ultrasonic scalers, and piezoelectric and magnetostrictive tips are widely used for the treatment of periodontitis and peri-implantitis. It has been demonstrated that the conventional mechanical instruments smooth the irregular surface of a diseased root, but they roughen the surface of a titanium implant [16,17,18,19]. To overcome the limitations of the metal devices, several alternative methods have been developed for the maintenance of dental implants, including plastic curettes, air-flow, rubber cups, and an ultrasonic system [20,21,22]. It has been demonstrated that the latter instruments cause little or no damage to smooth implant surfaces [23]. Moreover, abrasive air tools, such as airflow, are the devices of choice when it is required to maintain the integrity of the implant surface features [23]. Metal tools and cutters are recommended for smoothing the rough surface. However, some studies have highlighted their weaknesses, including the effects of inadequate fragility or toxicity of their residues [24,25]. Metal scalers produce defects in titanium implant surfaces; load and power are important factors in the damage caused. In a study conducted by Meschenmoser et al. in 1996, it was shown that the steel curette and the ultrasonic system were unsuitable for cleaning titanium implants [26]. On the other hand, plastic-coated scaler probes cause minimal damage to implant surfaces with a good polishing action but can leave plastic deposits behind on the implant surface [27]. Different methods of analysis were used to evaluate superficial topographic changes. In this sense, lacunarity analysis has been shown to be able to quantify distributions applied to data of any dimensionality and to provide information on structural changes within complex spatial structures [28]. 

The purpose of this study was to evaluate, by lacunarity analysis, the titanium surface alterations after treatment with two different types of ultrasonic tips: conventional steel and an innovative copper alloy silver-coated one.

## 2. Materials and Methods

The present study evaluated the following ultrasonic scaler tips: a conventional metal tip (EMS Electro Medical Sistem SA, Nyon, Switzerland) made of stainless steel and an Tip (B & L Biotech USA, Inc., Bala Cynwyd, PA, USA) consisting of a copper alloy core and an outer coating of silver. 

**Figure 1:**
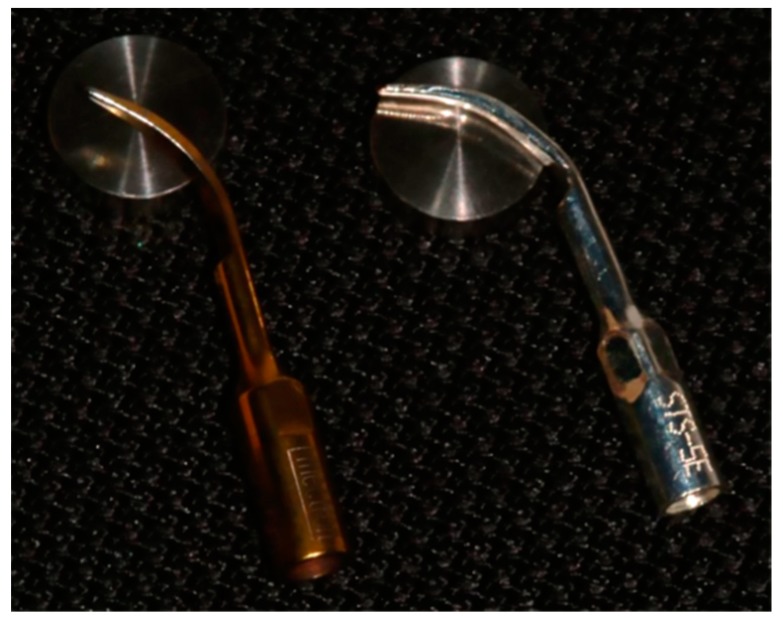
Photograph of conventional steel tip and copper alloy silver-plated tip on grade IV unalloyed titanium discs.

Twenty smooth-surface, grade IV unalloyed titanium discs, ISO 583/2 (GEASS s.r.l., Udine, Italy) with a diameter of 10 mm and height of 2.5 mm were divided into two groups (ten discs per group): Group A (conventional steel tips) and Group B (ultrasonic copper alloy silver plated tips) (Figure 1). After the surface preparation, these discs were washed in surfactant solution, treated with distilled water, and sterilized by gamma irradiation. The procedures for the evaluation of surface alteration were performed following a previous study [26]. Both types of tips were included in Mectron ultrasonic scalars (Mectron s.p.a., Carasco, Genova, Italy), fitted with a fixed angle of 45 degrees, using a custom-made swinging arm integrated with the recording equipment. The discs were in turn blocked in a container equipped with a safety valve for the custom-made piezoelectric water irrigation hand piece anchored stationary below the swinging arm. To obtain a constant calibration of the force, time, cycles, and the application tip’s angle on the titanium discs, a LR30K loading cell machine (Lloyd Instruments, part of AMTEK Test & Calibration Instruments, West Sussex, UK) with a primary code generated ad hoc for this study, was used. The primary code established that there were continuous cycles of the oscillating arm with an excursion fixed at 3 mm and with a speed of 1 mm/s, equivalent to that normally used in manual operation of the ultrasonic scalers. The cycle was completed in 20 s for each disc sample used.

The power of the scaler tips was set to intermediate according to the manufacturer’s advice. The surface morphology of each Ti disc was examined using a Scanning electron microscopy (SEM) (Carl Zeiss Evo 50, Oberkochen, Germany).

The discs were metallized by a gold-sputter Emitech K550 (Emitech Ltd., Ashford, UK) and subsequently inserted into the sample-holder for SEM analysis. The obtained images were then analysed using ImageJ software 1.48f 3D (Wayne Rasband, National Institutes of Health NIH, Bethesda, MD, USA), in order to highlight the incongruity of the surface in the individual discs and reconstruct a 3D faithful image of the same for further data collection for statistical analysis.

Statistical analysis was performed through one-way analysis of variance (ANOVA) and post hoc Scheffé test. All values were considered significant when *p* < 0.05. The fractal analysis of lacunarity (“VISUAL TEXTURE”) was performed by calculating the variation of the pixels’ number in each ε-sized box (CV) of a standard grid placed on SEM images and analysed through box counting overlap using the FracLac 2.5 Release 1d plugins of ImageJ 1.48f. The lacunarity was calculated from the standard deviation, σ, and mean, μ, for pixels per box (CV^2^). The value was collected for each ε in each series of grid sizes in a set of grid orientations. Briefly, the lacunarity count is based on variation in pixel density at different box sizes in fixed scans and sliding scans.

In order to perform these analyses the following algorithms were used:

$$\;\Lambda_{\epsilon }={\left(\sigma |\mu \right)}^{2}\;$$

$\Lambda_{\epsilon }\;$ is the visual texture scale (heterogeneity); (σ|μ) is the coefficient of variation (CV).

$$\;{\frac{\Sigma \left[\left(\frac{A^{-1}}{\frac{\Sigma \;A^{-1}}{N}}\right)-1\right]}{N}}^{2}\;$$

where *A* = is the pore factor; *N* = the number of grid locations.

Lacunarity analysis was used as a multiscale method for describing patterns of spatial dispersion. It can be used with both binary and quantitative data in one, two, and three dimensions. Although originally developed for fractal objects, the method is more general and can be readily used to describe nonfractal and multifractal patterns, such as the roughness evaluation of SEM images. The lacunarity analysis was based on studies of Plotnick [27], Mandelbrot [29], and Voss [30], which proved applicability of lacunarity analysis on various machining surfaces [31,32,33]. Group Tests have been performed to analyse the differences between groups. Student’s *t*-test for unpaired data was performed according to the method of the SPSS software (version 25, IBM, New Orchard Road Armonk, NY, USA). Levene’s Test was applied to verify that the difference is significant at the sample level, so as to extend the considerations to the entire population of the test samples. An independent statistician reviewed the methodology and statistical analysis. 

## 3. Results

**Figure 2:**
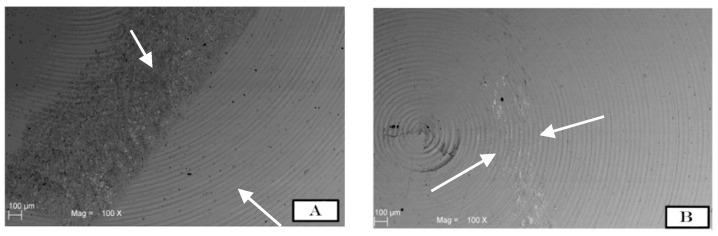
SEM images show how the titanium surface was altered by the use of steel tips (**A**) and by the new copper alloy silver-plated tip (**B**). Note how the irregularities are evident after the steel tip’s action on the disc surface, shown by the white arrows. On the other hand, image B shows how the surface is less altered in terms of surface topography modifications.

**Figure 3:**
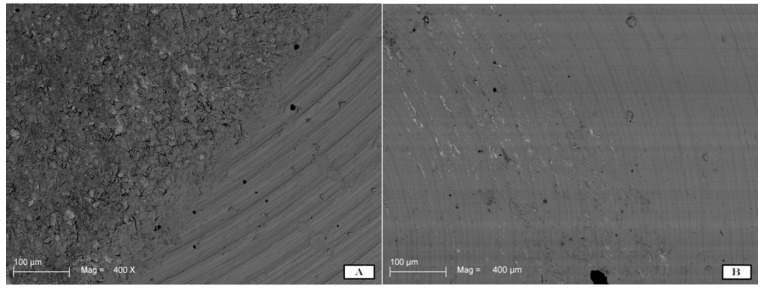
SEM images at higher magnification (400×) show the titanium surface alteration by the use of the steel tip (**A**) and by the new copper alloy silver-plated tip (**B**).

**Figure 4:**
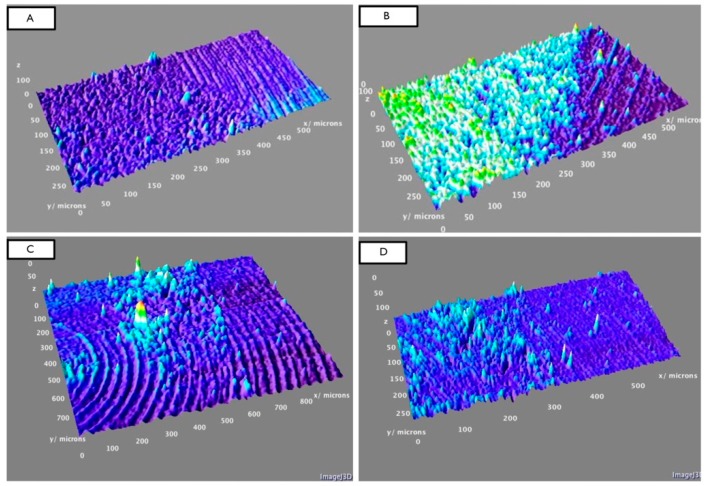
(**A**,**B**) show the 3D analysis of the surface roughness of titanium grade IV discs after using the conventional steel tip. Images (**C**,**D**) show the 3D analysis of the surface of the titanium grade 4 discs after using the new ultrasonic copper alloy silver-plated tip.

**Figure 5:**
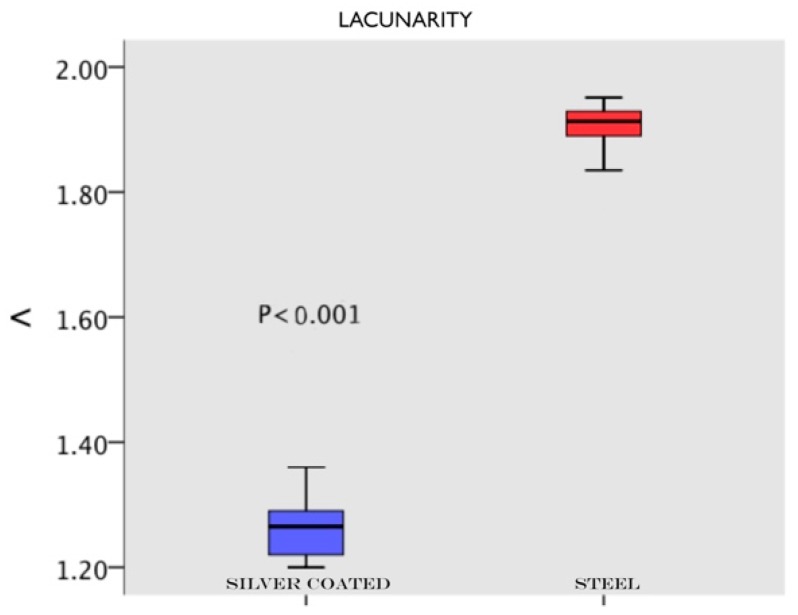
Graphical representation of fractal analysis of lacunarity where a statistical difference between groups is shown. Blue represents the new copper alloy silver-plated tip and red represents the conventional steel tip.

The SEM images were randomly taken in 5 different areas and analysed. The results demonstrated different degrees of surface roughness. In fact, the analysed surfaces showed clear differences between the two tips in terms of surface morphology alteration. (Figure 2A,B and Figure 3A,B) In particular, it emerged that the discs instrumented with the conventional steel tip showed important surface alterations. In contrast, samples treated with the copper alloy silver-plated ultrasonic tip had minimal structural changes on the surface. The SEM image of group A showed the presence of a deeper groove generated by the tip, compared with the one generated by the new tip from group B. In the same way, the superficial topography generated by the action of the tips evaluated by fractal analysis showed different results. Specifically, in Figure 4A–D, it is possible to see the 3D Fractal Dimension analysis of the titanium discs’ surface roughness after the use of the conventional steel tip (Figure 4A,B) and the ultrasonic copper alloy silver plated tip (Figure 4C,D). The measurements showed that the average roughness values obtained through fractal analysis of lacunarity (“VISUAL TEXTURE”) were 1.873 µm and 1.263 µm for group A and Group B, respectively. The statistical and fractal analysis showed a statistically significant difference between the two groups considered in this study, as shown in Table 1, confirming the SEM results. (Table 1, Figure 5).

## 4. Discussion

The use of dental implants represents an efficient option for the replacement of missing teeth. However, early and late complications, such as peri-implant infection or implant failure, can occur [34]. It has been demonstrated that peri-implantitis is associated with the presence of plaque and soft tissue inflammation [26]. Berglundh et al. suggested that the progression of peri-implantitis is more pronounced at implants with a moderately rough surface than at those with a polished surface [35]. Thus, strict periodontal control and decontamination of the implant surface is mandatory. Several studies have investigated and focused on surface alterations of titanium after instrumentation [21,36]. Simion et al. [37], in a 12-year retrospective study, investigated the long-term effectiveness of dental implants with a machined surface. A total of 59 implants were evaluated after 12 years of follow-up analyzing marginal bone loss around implants and clinical indices such as probing depth, bleeding on probing, and plaque score. At 12 years, no signs of peri-implantitis, such as bone loss, were present. Meanwhile, at this time point, clinical indices results showed moderate signs of soft tissue inflammation. Thus, the study demonstrated that a smooth surface could guarantee high success rates from a clinical point of view in a long-term follow-up [37]. Accordingly, the purpose of the present study was to investigate the surface alterations of machined titanium (Grade 4) discs with two different ultrasonic scaler tips. The study was conducted on non-surgical instrumentation normally used for implants maintenance in daily practice. The analysis of the two different ultrasonic inserts provided important results under scanning electron microscopy. In this regard, from the SEM analysis of the collected data, it was possible to analyse the surface conditions of the discs after tips use and therefore the impact caused by the two different types of ultrasonic tips.

In a recent study, mechanical instrumentation of novel metallic ultrasonic scaler tips, conventional stainless-steel tips, and plastic tips on titanium surfaces was evaluated on 10 polished commercially pure titanium discs (Grade II). Scanning electron microscopy (SEM) and commercial atomic force microscopy (AFM) were used to analyse the surface morphology of samples and the contact mode of the different tips [38]. It was concluded that the novel metallic copper alloy ultrasonic scaler tips minimally influence the titanium surface, similar to plastic tips. Therefore, they could be suitable instruments for implant maintenance [38,39,40]. These data were in accordance with the present study where the conventional tip demonstrated statistically significant surface morphology alterations compared to the new one, as shown in Figure 2 and Figure 3. On the other hand, to obtain a good level of debridement and to mechanically remove the biofilm on the implant surface, it is necessary to obtain good efficacy of the tips and, in the same way, minimal superficial alteration that could cause faster accumulation of plaque [41].

Another study evaluated two ultrasonic insert designs (metallic TFI-10 and a plastic-tipped implant insert) on titanium implants and evaluated their modified surfaces. The resulting of laser profilometry and scanning electron microscopy (SEM) showed that the metal scalers produced defects in the implant surfaces, with load and power being important factors in the damage caused. On the contrary, plastic-coated scaler probes cause minimal damage to implant surfaces and have a polishing action, but can leave plastic deposits on the implant surface [26].

Park et al. concluded that metal or plastic ultrasonic scaler tips may be applied to treat the sandblasted acid-etched (SLA) surface of dental abutment or fixture surfaces without increasing the irregularities on the titanium surfaces. However, in the case of machined surfaces, ultrasonic metal tips cannot be recommended because the surface becomes rougher after treatment [42].

The present results demonstrated that the new copper alloy silver-plated ultrasonic tip caused less damage to the polished surface that the conventional one. Irregularities on the titanium surface caused by the conventional tip consisted of shallower and deeper scratches than the new one. It has been demonstrated that surface irregularities can facilitate bacterial accumulation [5,43]. In this case the irregularities present on group A might provide a better niche for bacterial accumulation than those of group B. Therefore, careful instrumentation during implant maintenance is recommended. 

It is important to underline how the surfaces can be treated differently with different results in terms of cell adhesion and plaque accumulation [44,45]. Different authors studied predetermined roughness patterns by laser processing, obtaining good results in terms of cell adhesion. Sinjari et al. in 2012 showed how a controlled roughness surface treatment can increase osteoblastic cell growth with respect to sandblasted and machined surfaces [45]. Furthermore, controlled surface roughness was less attractive for bacterial colonization. Di Giulio et al. in 2016 [44] demonstrated by in vitro microbiological investigation how the biofilm formation of *Porphyromonas gingivalis* was reduced on laser-treated surfaces compared with sandblasted surfaces. In this case, even on a predetermined rough surface, bacterial accumulation was less than on the machined one, because of the laser treatment on the titanium surface. In fact, the authors think that the results are due to the bottom of the pores caused by the laser treatment, which resulted in a very polished surface that was not attractive for the bacteria. Meanwhile, in the complex, the laser treated surface is considered a rough one. Due to the above results, the surface is a key factor of bacterial attractiveness and rough surfaces with antibacterial proprieties could improve cell adhesion, while reducing bacterial plaque accumulation. The present results demonstrated the importance of implant maintenance instruments that do not affect the surface morphology of implants by making it irregular and more contaminable by bacteria. On the other hand, it is important to see the decontamination effects of the two present scaler tips on in vivo plaque accumulation.

Therefore, other investigations are necessary to identify the best surface treatment in terms of cell adhesion and reduced bacterial colonization. It has been proven that the surface alterations caused by decontamination operations can alter the surface, thus reducing its biocompatibility and altering its surface characteristics. In fact, other studies showed how traditional tips could alter surface roughness, leave traces during instrumentation, and remove material from the surface, regardless of surface treatment. So, the tested tip seems to have a less-damaging effect on the implant surface, thus reducing the destructive effects of decontamination procedures [19,46].

Moreover, it has been demonstrated that restorative dental materials, such as porcelain or composite resins, if instrumented with power-driven scalers, may experience chips, scratches, or loss of material. Indeed, current evidence suggests that operation of ultrasonic scalers at medium rather than high power may cause less damage to implant surfaces [36,47,48,49,50]. 

In this study, standardized conditions including angulation of the scaler tip at 45 degrees, medium power, and irrigation were used to reproduce clinical conditions.

However, future research is necessary to investigate the clinical significance of the new copper alloy silver-plated ultrasonic scaler tip regarding surface alterations during peri-implant treatment in human subjects under standard clinical conditions.

## 5. Conclusions

Within the limitations of the present study, the following conclusions were drawn. Surface alterations of titanium induced by the conventional ultrasonic tips were much greater than those made by copper alloy silver plated tips These results are an interesting topic for further in vivo and/or microbiological analysis to evaluate the removal efficiency of the copper alloy silver-plated tips and to encourage their use for implant maintenance.

## Figures and Tables

**Table 1:** Group Tests and tests for Independent Samples showed a statistically significant difference between the two groups in terms of lacunarity (*p* < 0.05). Student’s *t* test for unpaired data and Levene’s Test were performed to verify the significance of the difference between groups.

**Group Members**	**Group**		**N**		**Mean (St. Dev)**			**St. Error**	
A	Steel		10		1.8736 (0.08767)			0.02772	
B	Silver Coated		10		1.2630 (0.05034)			0.01592	
**Statistical Analysis**	**Levene’s Test Used to Assess the Equality of Variances**		***t*-test Used for Comparison of Means**						
	F	Sig.	t	df	Sig. (2-code)	Difference between means	Difference Standard error	a 95% confidence interval	
								Inferior	Superior
Equal variances	2.149	0.160	19.099	18	0.000	0.61060	0.03197	0.54343	0.67777
Non-equal variances			19.099	14.353	0.000	0.61060	0.03197	0.54219	0.67901

## References

[1] Zitzmann N.U., Berglundh T. (2008). Definition and prevalence of peri-implant diseases. J. Clin. Periodontol..

[2] Ramanauskaite A., Becker K., Schwarz F. (2018). Clinical characteristics of peri-implant mucositis and peri-implantitis. Clin. Oral Implants Res..

[3] Scarano A., Piattelli A., Quaranta A., Lorusso F. (2017). Bone Response to Two Dental Implants with Different Sandblasted/Acid-Etched Implant Surfaces: A Histological and Histomorphometrical Study in Rabbits. Biomed. Res. Int..

[4] Sinjari B., Guarnieri S., Diomede F., Merciaro I., Mariggio M.A., Caputi S., Trubiani O. (2012). Influence of titanium laser surface geometry on proliferation and on morphological features of human mandibular primary osteoblasts. J. Biol. Regul. Homeost. Agents.

[5] Simion M., Kim D.M., Pieroni S., Nevins M., Cassinelli C. (2016). Bacterial Biofilm Morphology on a Failing Implant with an Oxidized Surface: A Scanning Electron Microscope Study. Int. J. Periodontics Restorative Dent..

[6] Drago L., Bortolin M., De Vecchi E., Agrappi S., Weinstein R.L., Mattina R., Francetti L. (2016). Antibiofilm activity of sandblasted and laser-modified titanium against microorganisms isolated from peri-implantitis lesions. J. Chemother..

[7] Renvert S., Roos-Jansåker A.M., Claffey N. (2008). Non-surgical treatment of peri-implant mucositis and peri-implantitis: A literature review. J. Clin. Periodontol..

[8] Jepsen S., Berglundh T., Genco R., Aass A.M., Demirel K., Derks J., Figuero E., Giovannoli J.L., Goldstein M., Lambert F. (2015). Primary prevention of peri-implantitis: Managing peri-implant mucositis. J. Clin. Periodontol..

[9] Esposito M., Grusovin M.G., Worthington H.V. (2012). Treatment of peri-implantitis: What interventions are effective? A Cochrane systematic review. Eur. J. Oral Implantol..

[10] Persson L.G., Berglundh T., Lindhe J., Sennerby L. (2001). Re-osseointegration after treatment of peri-implantitis at different implant surfaces. An experimental study in the dog. Clin. Oral Implants Res..

[11] Kuempel D.R., Johnson G.K., Zaharias R.S., Keller J.C. (1995). The effects of scaling procedures on epithelial cell growth on titanium surfaces. J. Periodontol..

[12] Smeets R., Henningsen A., Jung O., Heiland M., Hammächer C., Stein J.M. (2014). Definition, etiology, prevention and treatment of peri-implantitis—A review. Head Face Med..

[13] Elemek E., Almas K. (2014). Peri-implantitis: Etiology, diagnosis and treatment: An update. J. Clin. Periodontol..

[14] Robertson K., Shahbazian T., MacLeod S. (2015). Treatment of peri-implantitis and the failing implant. Dent. Clin. North Am..

[15] Pranskunas M., Poskevicius L., Juodzbalys G., Kubilius R., Jimbo R. (2016). Influence of Peri-Implant Soft Tissue Condition and Plaque Accumulation on Peri-Implantitis: A Systematic Review. J. Oral Maxillofac. Res..

[16] Augthun M., Tinschert J., Huber A. (1998). In vitro studies on the effect of cleaning methods on different implant surfaces. J. Periodontol..

[17] Menezes K.M., Fernandes-Costa A.N., Silva-Neto R.D., Calderon P.S., Gurgel B.C. (2016). Efficacy of 0.12% Chlorhexidine Gluconate for Non-Surgical Treatment of Peri-Implant Mucositis. J. Periodontol..

[18] Ziebolz D., Klipp S., Schmalz G., Schmickler J., Rinke S., Kottmann T., Fresmann S., Einwag J. (2017). Comparison of different maintenance strategies within supportive implant therapy for prevention of peri-implant inflammation during the first year after implant restoration. A randomized, dental hygiene practice-based multicenter study. Am. J. Dent..

[19] Mengel R., Buns C.E., Mengel C., Flores-de-Jacoby L. (1998). An in vitro study of the treatment of implant surfaces with different instruments. Int. J. Oral Maxillofac. Implants.

[20] Barnes C.M., Fleming L.S., Mueninghoff L.A. (1991). SEM evaluation of the in–vitro effects of an air-abrasive system on various implant surfaces. Int. J. Oral Maxillofac. Implants.

[21] Kawashima H., Sato S., Kishida M., Ito K. (2007). A comparison of root surface instrumentation using two piezoelectric ultrasonic scalers and a hand scaler in vivo. J. Periodontal Res..

[22] Trejo P.M., Bonaventura G., Weng D., Caffesse R.G., Bragger U., Lang N.P. (2006). Effect of mechanical and antiseptic therapy on peri-implant mucositis: An experimental study in monkeys. Clin. Oral Implants Res..

[23] Louropoulou A., Slot D.E., Van der Weijden F.A. (2012). Titanium surface alterations following the use of different mechanical instruments: A systematic review. Clin. Oral Implants Res..

[24] Schwarz F., Ferrari D., Popovski K., Hartig B., Becker J. (2009). Influence of different air-abrasive powders on cell viability at biologically contaminated titanium dental implants surfaces. J. Biomed. Mater. Res. B. Appl. Biomater..

[25] Schwarz F., Rothamel D., Sculean A., Georg T., Scherbaum W., Becker J. (2003). Effects of an Er: YAG laser and the Vector ultrasonic system on the biocompatibility of titanium implants in cultures of human osteoblast-like cells. Clin. Oral Implants Res..

[26] Meschenmoser A., d’Hoedt B., Meyle J., Elssner G., Korn D., Hämmerle H., Schulte W. (1996). Effects of various hygiene procedures on the surface characteristics of titanium abutments. J. Periodontol..

[27] Mann M., Parmar D., Walmsley A.D., Lea S.C. (2012). Effect of plastic-covered ultrasonic scalers on titanium implant surfaces. Clin. Oral Implants Res..

[28] Plotnick R.E., Gardner R.H., Hargrove W.W., Prestegaard K., Perlmutter M. (1996). Lacunarity analysis: A general technique for the analysis of spatial patterns. Phys. Rev. E.

[29] Mandelbrot B. (1982). The Fractal Geometry of Nature.

[30] Voss R.F., Pynn R., Skjeltorp A. (1991). Random Fractals: Characterization and measurement. Scaling Phenomena in Disordered Systems.

[31] Bojović B., Koruga Đ. (2012). Micro and nano lubricant behavior of tear film aqueous layer. Contemp. Mater..

[32] Tomic M., Bojovic B., Stamenkovic D., Mileusnic I., Koruga Đ. (2017). Lacunarity Properties of Nanophotonic Materials Based on Poly(Methyl Methacrylate) for Contact Lenses. Mater. Technol..

[33] Hoechstetter S., Walz U., Thinh N. (2011). Adapting lacunarity techniques for gradient-based analyses of landscape surfaces. Ecol. Complex..

[34] Tey V.H.S., Phillips R., Tan K. (2017). Five-year retrospective study on success, survival and incidence of complications of single crowns supported by dental implants. Clin. Oral Implants Res..

[35] Berglundh T., Gotfredsen K., Zitzmann N.U., Lang N.P., Lindhe J. (2007). Spontaneous progression of ligature induced peri-implantitis at implants with different surface roughness: An experimental study in dogs. Clin. Oral Implants Res..

[36] Ramaglia L., di Lauro A.E., Morgese F., Squillace A. (2006). Profilometric and standard error of the mean analysis of rough implant surfaces treated with different instrumentations. Implant Dent..

[37] Simion M., Gionso L., Grossi G.B., Briguglio F., Fontana F. (2015). Twelve-Year Retrospective Follow-Up of Machined Implants in the Posterior Maxilla: Radiographic and Peri-Implant Outcome. Clin. Implant Dent. Relat. Res..

[38] Baek S.H., Shon W.J., Bae K.S., Kum K.Y., Lee W.C., Park Y.S. (2012). Evaluation of the safety and efficiency of novel metallic ultrasonic scaler tip on titanium surfaces. Clin. Oral Implants Res..

[39] Mellado-Valero A., Buitrago-Vera P., Solá-Ruiz M.F., Ferrer-García J.C. (2013). Decontamination of dental implant surface in peri-implantitis treatment: A literature review. Med. Oral Patol. Oral Cir. Bucal..

[40] Subramani K., Wismeijer D. (2012). Decontamination of titanium implant surface and re-osseointegration to treat peri-implantitis: A literature review. Int. J. Oral Maxillofac. Implants..

[41] Matarasso S., Quaremba G., Coraggio F., Vaia E., Cafiero C., Lang N.P. (1996). Maintenance of implants: An in vitro study of titanium implant surface modifications subsequent to the application of different prophylaxis procedures. Clin. Oral Implants Res..

[42] Park J.B., Kim N., Ko Y. (2012). Effects of ultrasonic scaler tips and toothbrush on titanium disc surfaces evaluated with confocal microscopy. J. Craniofac. Surg..

[43] Quirynen M., Vogels R. (2002). Clinical relevance of surface characteristics on the formation of plaque on teeth and implants. Ned. Tijdschr. Tandheelkd..

[44] Di Giulio M., Traini T., Sinjari B., Nostro A., Caputi S., Cellini L. (2016). Porphyromonas gingivalis biofilm formation in different titanium surfaces, an in vitro study. Clin. Oral Implants Res..

[45] Sinjari B., Traini T., Caputi S., Mortellaro C., Scarano A. (2018). Evaluation of Fibrin Clot Attachment on Titanium Laser-Conditioned Surface Using Scanning Electron Microscopy. J. Craniofac. Surg..

[46] Rapley J.W., Swan R.H., Hallmon W.W., Mills M.P. (1990). The surface char- acteristics produced by various oral hygiene instruments and mate- rials on titanium implant abutments. Int. J. Oral Maxillofac. Implants.

[47] Fox S.C., Moriarty J.D., Kusy R.P. (1990). The effects of scaling a titanium implant surface with metal and plastic instruments: An in vitro study. J. Periodontol..

[48] Mengel R., Meer C., Flores-de-Jacoby L. (2004). The treatment of uncoated and titanium nitride-coated abutments with different instruments. Int. J. Oral Maxillofac. Implants.

[49] Bailey G.M., Gardner J.S., Day M.H., Kovanda B.J. (1998). Implant surface alterations from a nonmetallic ultrasonic tip. J. West. Soc. Periodontol. Periodontal Abstr..

[50] Tawse-Smith A., Atieh M.A., Tompkins G., Duncan W.J., Reid M.R., Stirling C.H. (2016). The effect of piezoelectric ultrasonic instrumentation on titanium discs: A microscopy and trace elemental analysis in vitro study. Int. J. Dent. Hyg..

